# Choline PET based dose-painting in prostate cancer - Modelling of dose effects

**DOI:** 10.1186/1748-717X-5-23

**Published:** 2010-03-18

**Authors:** Maximilian Niyazi, Peter Bartenstein, Claus Belka, Ute Ganswindt

**Affiliations:** 1 Department of Radiation Oncology, Ludwig-Maximilians-University München, Marchioninistr. 15, 81377 München, Germany; 2 Department of Nuclear Medicine, Ludwig-Maximilians-University München, Marchioninistr. 15, 81377 München, Germany

## Abstract

**Background:**

Several randomized trials have documented the value of radiation dose escalation in patients with prostate cancer, especially in patients with intermediate risk profile. Up to now dose escalation is usually applied to the whole prostate. IMRT and related techniques currently allow for dose escalation in sub-volumes of the organ. However, the sensitivity of the imaging modality and the fact that small islands of cancer are often dispersed within the whole organ may limit these approaches with regard to a clear clinical benefit. In order to assess potential effects of a dose escalation in certain sub-volumes based on choline PET imaging a mathematical dose-response model was developed.

**Methods:**

Based on different assumptions for α/β, γ50, sensitivity and specificity of choline PET, the influence of the whole prostate and simultaneous integrated boost (SIB) dose on tumor control probability (TCP) was calculated. Based on the given heterogeneity of all potential variables certain representative permutations of the parameters were chosen and, subsequently, the influence on TCP was assessed.

**Results:**

Using schedules with 74 Gy within the whole prostate and a SIB dose of 90 Gy the TCP increase ranged from 23.1% (high detection rate of choline PET, low whole prostate dose, high γ50/ASTRO definition for tumor control) to 1.4% TCP gain (low sensitivity of PET, high whole prostate dose, CN + 2 definition for tumor control) or even 0% in selected cases. The corresponding initial TCP values without integrated boost ranged from 67.3% to 100%. According to a large data set of intermediate-risk prostate cancer patients the resulting TCP gains ranged from 22.2% to 10.1% (ASTRO definition) or from 13.2% to 6.0% (CN + 2 definition).

**Discussion:**

Although a simplified mathematical model was employed, the presented model allows for an estimation in how far given schedules are relevant for clinical practice. However, the benefit of a SIB based on choline PET seems less than intuitively expected. Only under the assumption of high detection rates and low initial TCP values the TCP gain has been shown to be relevant.

**Conclusions:**

Based on the employed assumptions, specific dose escalation to choline PET positive areas within the prostate may increase the local control rates. Due to the lack of exact PET sensitivity and prostate α/β parameter, no firm conclusions can be made. Small variations may completely abrogate the clinical benefit of a SIB based on choline PET imaging.

## Introduction

Several randomized trials have documented a clear dose-response relationship for prostate cancer. Although not employing modern IMRT techniques the M. D. Anderson phase III dose escalation trial was the first randomized trial to prove 78 Gy vs. 70 Gy. It resulted in better biochemical control for the higher radiation dose in patients with intermediate-risk features [1]. Other groups obtained similar results [2-6]. This interpretation is corroborated by population based approaches showing that only doses ≥ 72 Gy are associated with adequate tumor control [7,8].

The implementation of IMRT into clinical practice of prostate cancer radiation treatment enables the physician to increase the doses in focal areas of the gland, which is in contrast to the central dogma in radiation oncology to strive for a homogeneous dose to the target volume [9]. However, this approach might have two advantages: Firstly the dose escalation is limited to a minor part of the target volume and thus, the probability of side effects should be lowered [10]. Secondly the biological efficacy may be increased by the use of higher doses per fraction.

The first who addressed this issue were Pickett, Xia and colleagues [11,12], later on further studies were conducted [13,14], also in case of high-risk prostate cancer [15]. Li et al. reported a new IMRT simultaneous integrated boost (SIB) strategy that irradiates prostate via hypo-fractionation while irradiating pelvic nodes with the conventional fractionation. Compared to the conventional two-phase treatment, the proposed SIB technique offers potential advantages, including better sparing of critical structures leading to less incontinence, rectal bleeding, irritative symptoms [16-20] or urethral toxicity [21], more efficient delivery, shorter treatment duration, and better biological efficacy [22]. Fonteyne et al. reported that addition of an IMRT SIB to an intra-prostatic lesion (defined by magnetic resonance imaging) did not increase the severity or incidence of acute toxicity [23]. Furthermore new techniques like volumetric modulated arcs, helical tomotherapy or IMPT additionally showed improvements in conformal avoidance relative to fixed beam IMRT [24,25].

Despite the technical advances in radiotherapy the optimal treatment for prostate cancer strongly depends on the accuracy of tumor characterization and staging. Positron emission tomography (PET) is an exquisitely sensitive molecular imaging technique using positron-emitting radioisotopes coupled to specific ligands [26].

Different PET tracers, including [^11^C] choline, [^18^F] choline and [^11^C] acetate, have been described for the detection of prostate cancer. However, larger trials are still needed to establish their final clinical value concerning the primary detection and the staging of prostate cancer [27].

In principle, signal-generation is based on an increased choline metabolism in prostate cancer leading to an increased up-take in tumor tissue compared to that of benign tissue [28]. However, benign prostate hyperplasia and inflammatory changes may also lead to increased uptake thereby lowering the specificity of the PET signal.

A precise volumetric assessment of PET signals is of rising importance for radiotherapy (RT) planning [29]. The use of choline PET/CT data to detect tumor spots within the prostate has been analyzed and first clinical experiences in lymph node-positive patients were reported [30]. In this regard, Ciernik et al. investigated the utility of F-18-choline PET signals to serve as a target for semi-automatic segmentation for forward treatment planning of prostate cancer. F-18-choline PET and CT scans of ten patients with histologically proven prostate cancer without extra-capsular tumor extension were acquired using a combined PET/CT scanner. Planning target volumes (PTV's) derived from CT and F-18-choline PET yielded comparable results. 3D-conformal planning with CT or F-18-choline PET resulted in comparable doses to the rectal wall. Choline PET signals of the prostate provided adequate spatial information to be used for standardized PET-based target volume definition [31].

As PET allows for detection of small lesions within the prostate and modern IMRT techniques can be used for integrated focal boosting, it is evident to use PET information in order to escalate the dose within defined tumor spots also called biologically guided radiotherapy [32]. This type of selective dose-escalation has already been implemented successfully using spectroscopic MRI data [23,33,34]. Although doing so may be intuitively reasonable, the true effect of such procedures is strongly influenced by a multitude of factors. We therefore attempted to develop a method to estimate the increase of local tumor control using an IMRT SIB to choline PET positive hotspots within the gland. The computations were done in a putative intermediate-risk collective reflecting the fact that these patients will have the most benefit by any dose escalation approach.

## Methods

The best currently available dataset for dose-response relationships in prostate cancer was derived from a study of 235 low-risk and 382 intermediate-risk patients treated between 1987 and 1998 with external beam RT alone at the M. D. Anderson Cancer Center [35].

Local control (biochemical no evidence of disease) was defined in two different ways; Firstly, ASTRO definition was employed: Time to PSA failure is defined as the end of RT to the mid-point between the PSA nadir and the first PSA rise [35]. Secondly, the Houston definition defines biochemical failure as PSA rise of ≥ 2 ng/ml above the current nadir PSA (CN + 2) [36-38]. In both settings detectable local, nodal and distant relapses as well as initiation of hormonal treatment are scored as failures.

In order to develop a mathematical TCP model for prostate cancer, we firstly assumed the prostate to be a geometrical structure subdivided into a fixed number of voxels (defining their volume as v_i _= 1). Voxels including tumor cells are called tumorlets.

N is defined as the number of clonogenic cells within the tumor, V as the volume of the target volume and n_i _is defined as the density of tumor cells within a tumorlet. We furthermore assumed that all tumorlets have the same density of clonogenic cells. In order to achieve this in practice one has to define the voxels as sufficiently small.

The tumor control probability (TCP) is modelled as a Poisson distribution [39]. In such a geometrical setting it is defined as:

SF_i _is the surviving fraction within the single sub-volume with the running index i (ranging from 1 to m = V/v_i_). Using the well-known linear-quadratic model the surviving fraction can be calculated as:

with d_j _as single dose (usually 1.8 or 2 Gy), n as the number of fractions and α, β as the parameters from the linear-quadratic model which refer to the radio-sensitivity of the tumor cells (α represents lethal lesions made by one-track action and β accounts for lethal lesions made by two-track action, [40]). In this formula the tumor doubling time is not considered.

Relevant α/β ratios can be obtained from both in vitro experiments and clinical fractionation studies and give the dose where linear and quadratic effect are equal according to total cell kill [41] whereas in vitro data do not necessarily predict the radio-sensitivity of tissues in clinical radiotherapy. There is a wide variation of α/β values for prostate cancer in the literature with the exact value of α/β being still unknown [41-51].

Thus, the following calculations were based on the values determined by Fowler et al. (α/β = 1.5 Gy, α = 0.04 Gy^-1^) [43], Wang et al. (α/β = 3.1 Gy, α = 0.15 Gy^-1 ^[49,52]) and Valdagni et al. (α/β = 8.3 Gy [46,48]).

**Figure 1:**
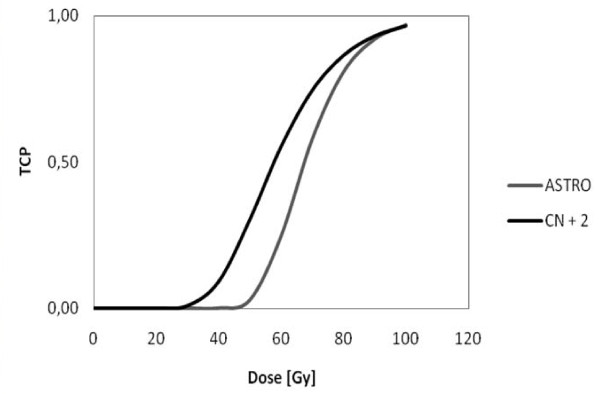
**Tumor control probability curves for both definitions of local control derived by data of Cheung et al**. (RT of the whole prostate).

Another relevant parameter to describe the TCP is the slope of the killing curve (γ50) which relates to the number of clonogens within the tumor in the following way [53]:

Cheung et al. calculated a γ50 value of 2.2 [1.1-3.2, 95% CI] and TCD50 = 67.5 Gy [65.5-69.5 Gy, 95% CI] (ASTRO definition) or γ50 = 1.4 [0.2-2.5, 95% CI] and TCD50 = 57.8 Gy [49.8-65.9 Gy, 95% CI] (CN + 2 definition) for intermediate-risk patients [35]. The corresponding TCP curves are shown in Figure 1.

Those voxels not containing a clonogenic cell (pure prostate tissue) do not contribute to the overall TCP as the corresponding factor equals 1.

Summarizing all these equations, and after some algebraic manipulations keeping in mind that v_i _= 1, one obtains:

ΔSF denotes the difference between boosted and conventional surviving fraction (conventional means without boost, but 3D-conformal RT or IMRT technique). This expression has to be corrected due to the limited sensitivity in detecting all clonogenic cells. The sensitivity values for choline PET range from 81% (for a SUV of 2.65) [54] down to 73% [28,55] or 64% [56] (Additional file 1 offers the possibility to specify different parameters for intermediate-risk prostate cancer to calculate the effect of an IMRT SIB).

This is a simplified picture of reality as the sensitivity of detecting tumor cells within the prostate is dependent on the size or more precise intensity of the enhancing tumor lesion. Partial volume effects can severely affect images both qualitatively and quantitatively: For any hot lesion of a small size and embedded in a colder background, this effect spreads out the signal. It typically occurs whenever the tumor size is less than 3 times the full width at half maximum (FWHM) of the reconstructed image resolution. The maximum value in the hot tumor then will be lower than the actual maximum value. A small tumor will look larger but less aggressive than it actually is [57]. The model assumes the detection rate for the sake of simplicity size-independent and constant, the aforementioned sensitivities from the literature are taken as best guesses for the detection rate.

The model used for our calculation is based on a number of additional assumptions. Thus, several shortcomings have to be taken into account when interpreting the data:

1) The assumption of a homogeneous density of clonogenic tumor-cells is not obvious. There may be islands within the prostate with a higher clonogenic density. However, this is no strict contradiction to our assumption as the sub-voxels may be scaled down until only empty voxels and voxels with a small but uniform number of clonogenic cells remain left.

2) The given model is incapable of reflecting biological sub-volume effects adequately: For example, one may assume that hypoxic areas within high-density tumor foci may cause a locally enhanced radio-resistance. Since all values used for our calculation are based on whole organ TCPs, the given model ignores issues of focally increased resistance.

3) Biologically, a complex feedback between the tumor and surrounding normal tissue exists. For example, the release of certain cytokines after radiation damage may influence the surrounding tumor tissue and vice versa. Again the given model is not able to integrate the putative interaction of adjacent clonogenic tumor and stroma cells.

4) It is assumed that all clonogenic cells within the tumor have a uniform radiosensitivity.

All these effects may be in place but do not seem to have much influence in practice. One prominent example is the comparison between primary and salvage radiotherapy.

After prostatectomy with positive surgical margins adjuvant radiotherapy improves disease-free survival rates and thus it is discussed as a new standard of adjuvant treatment in selected cases [58]; in cases of local relapse, salvage radiotherapy is the only potentially curative treatment approach [59]. The doses being necessary to control microscopic tumor seem to be higher than initially expected and to be similar to those for macroscopic tumor within the setting of a primary treatment [60].

## Results

The relevant parameters fed into our model in order to calculate the increase in whole organ TCP are: Sensitivity of choline PET, α, α/β, γ50, whole prostate dose, SIB dose and dose per fraction.

In order to present the calculations different representative scenarios have been tested:

### 1. High sensitivity of choline PET, low whole prostate dose, high γ50 (ASTRO consensus), Fowler's α/β

This parameter set was chosen to calculate a putative maximum TCP increase: Choline PET sensitivity was set to 81% and 74 Gy were chosen as homogeneous prostate dose. α/β was set to 1.5 Gy (α = 0.04 Gy^-1^), γ50 was chosen according to Cheung's data with the ASTRO definition. As shown in Figure 1 this parameter set leads to a higher steepness of the TCP curve. The results are shown in Table 1. The TCP in this setting with homogeneous dose of 74 Gy within the prostate was 67.3% and was improved by 23.1% up to 90.4% using a SIB.

**Table 1:** TCP-increase for high sensitivity of choline PET, low whole prostate dose, high γ50(ASTRO consensus) and Fowler's α/β

α [Gy^-1^]	α/β [Gy]	γ50	Det. rate PET [%]	Dose [Gy]	SIB [Gy]	Single dose [Gy]	TCP_conv _[%]	TCP Increase [%]
**0.04**	1.5	2.2	81	74	90	2	67.3	23.1

Calculation of the increase in TCP with whole prostate dose of 74 Gy after boosting choline PET positive regions within the prostate up to 90 Gy. α and α/β are estimated from Fowler's data and γ50 from Cheung's data (ASTRO definition). For choline PET a high sensitivity was used.

### 2. High sensitivity of choline PET, low whole prostate dose, low γ50 (CN + 2 definition), Fowler's α/β

In contrast, one may assume a parameter set with slightly less optimal conditions for a SIB. Table 2 summarizes the results when assuming a higher detection rate for PET (81%), a low homogeneous whole prostate dose (74 Gy), a SIB dose of 90 Gy and radio-sensitivity parameters as described by Fowler et al. (α/β = 1.5 Gy, α = 0.04 Gy^-1^) and γ50 taken again from Cheung's data but this time according to the CN + 2 definition. The calculated TCP without SIB was 96.0% which leaves only an increase of 2.9% with a SIB.

**Table 2:** TCP-increase for high sensitivity of choline PET, low whole prostate dose, low γ50(CN + 2 definition) and Fowler's α/β

α [Gy^-1^]	α/β [Gy]	γ50	Det. rate PET [%]	Dose [Gy]	SIB [Gy]	Single dose [Gy]	TCP_conv _[%]	TCP Increase [%]
0.04	1.5	1.4	81	74	90	2	96.0	2.9

Calculation of the TCP-increase after boosting choline PET positive regions within the prostate up to 90 Gy. α/β is estimated from Fowler's data and γ50 from Cheung's data (CN + 2 definition). For choline PET a high sensitivity was used.

This result is basically driven by a high initial control probability. In reality the initial clinical control probability is lower [35].

### 3. Low sensitivity of choline PET, high whole prostate dose, low γ50 (CN + 2 definition), Fowler's α/β

A "worst case" scenario is considered where a low sensitivity of PET is presumed, the homogeneous whole prostate dose is high (see Table 3, 78 Gy along the dose concept of the M. D. Anderson trial [1]), α/β is low and γ50 is less steep than the corresponding ASTRO value.

**Table 3:** TCP-increase for low sensitivity of choline PET, high whole prostate dose, low γ50(CN + 2 definition) and Fowler's α/β

α [Gy^-1^]	α/β [Gy]	γ50	Det. rate PET [%]	Dose [Gy]	SIB [Gy]	Single dose [Gy]	TCP_conv _[%]	TCP Increase [%]
0.04	1.5	1.4	64	78	90	2	97.0	1.4

Calculation of the TCP-increase after boosting choline PET positive regions within the prostate up to 90 Gy; the whole organ dose was set to 78 Gy. α/β is estimated from Fowler's data and γ50 from Cheung's data (CN + 2 definition). For choline PET a low sensitivity was used.

Based on these assumptions the gain of a SIB is low, as the initial TCP is again very high (97.0%) and as the remaining SIB effect is small (1.4%). Again, this result is in contrast to clinical reality reflected in the Cheung data [35].

### 4. High sensitivity of choline PET, low whole prostate dose, γ50 arbitrary, Wang's α/β

Using α/β and α values originally obtained by Wang et al. one obtains independently of γ50 or the whole organ dose a TCP of 100% which leaves no benefit for a SIB (Table 4). This result is probably due to the fact that the respective γ50 as well as α/β parameters were derived from independent clinical trials.

**Table 4:** TCP-increase for high sensitivity of choline PET, low whole prostate dose, γ50arbitrary and Wang's α/β

α [Gy^-1^]	α/β [Gy]	γ50	Det. rate PET [%]	Dose [Gy]	SIB [Gy]	Single dose [Gy]	TCP_conv _[%]	TCP Increase [%]
0.15	3.1	1.4	81	74	90	2	100	0
0.15	3.1	2.2	81	74	90	2	100	0

Calculation of the TCP-increase after boosting choline PET positive regions within the prostate up to 90 Gy. α/β is estimated from Wang's data and γ50 arbitrary. For choline PET a high sensitivity was assumed.

### 5. Different sensitivities of choline PET, low whole prostate dose, different α/β values, calculated α, high γ50 (ASTRO definition)

In order to circumvent the problem of overestimating the initial TCP one can try to reproduce the M. D. Anderson data (Cheung et al.) employing different α/β values (Fowler, Wang, Valdagni) and fitting an optimal α value to finally achieve a realistic concordance between observed TCD50 and calculated TCD50 value.

In Table 5 the ASTRO consensus was used for the definition of tumor control, leading to a steeper TCP curve (see Figure 1). Using a low whole prostate dose (74 Gy), the baseline tumor control was 68.7%. In this setting the SIB mediated TCP increase was strongly dependent on the sensitivity of the choline PET. Assuming a sensitivity rate of 81%, the TCP was increased by 22.2%, for 64% the increase was lowered to 17.0%.

**Table 5:** TCP-increase for different sensitivities of choline PET, low whole prostate dose, different α/β values, calculated α and high γ50 (ASTRO definition)

α [Gy^-1^]	α/β [Gy]	γ50	Det. rate PET [%]	Dose [Gy]	SIB [Gy]	Single dose [Gy]	TCP_conv _[%]	TCP Increase [%]
0.04	1.5	2.2	81	74	90	2	68.7	22.2
0.04	1.5	2.2	64	74	90	2	68.7	17.0
0.06	3.1	2.2	81	74	90	2	68.7	21.4
0.06	3.1	2.2	64	74	90	2	68.7	16.4
0.08	8.3	2.2	81	74	90	2	68.7	20.1
0.08	8.3	2.2	64	74	90	2	68.7	15.4

Calculation of the TCP-increase after boosting choline PET positive regions within the prostate up to 90 Gy. α/β was set to either Fowler's/Wang's or Valdagni's value, α was analytically determined in order to achieve agreement between calculated TCD50 and TCD50 obtained by Cheung et al. γ50 was again taken from Cheung's data (ASTRO definition).

Using higher α/β values automatically resulted in a lower TCP gain. This difference is based on the fact that in the given model α was optimized with fixed γ50 and α/β, resulting in different TCP curves.

### 6. Different sensitivities of choline PET, high whole prostate dose, different α/β values, calculated α, high γ50 (ASTRO definition)

Compared to Table 5 in Table 6 a higher whole prostate dose (78 Gy) was used. The initial TCP could be improved to 77.2%. The increase in TCP by the given SIB dose was lower ranging from 14.9% (high PET sensitivity, low α/β) to 10.1% (low detection rate, high α/β).

**Table 6:** TCP-increase for different sensitivities of choline PET, high whole prostate dose, different α/β values, calculated α and high γ50(ASTRO definition)

α [Gy^-1^]	α/β [Gy]	γ50	Det. rate PET [%]	Dose [Gy]	SIB [Gy]	Single dose [Gy]	TCP_conv _[%]	TCP Increase [%]
0.04	1.5	2.2	81	78	90	2	77.2	14.9
0.04	1.5	2.2	64	78	90	2	77.2	11.5
0.06	3.1	2.2	81	78	90	2	77.2	14.1
0.06	3.1	2.2	64	78	90	2	77.2	11.0
0.08	8.3	2.2	81	78	90	2	77.2	13.1
0.08	8.3	2.2	64	78	90	2	77.2	10.1

Calculation of the TCP-increase after boosting choline PET positive regions within the prostate up to 90 Gy, higher homogeneous whole prostate dose. α/β was analytically determined in order to achieve agreement between calculated TCD50 and TCD50 obtained by Cheung et al. γ50 was again taken from Cheung's data (ASTRO definition).

### 7. Different sensitivities of PET, low whole prostate dose, different α/β values, calculated α, low γ50 (CN + 2 definition)

In Table 7 the CN + 2 consensus was used to define tumor control, leading to less steep TCP curves (see Figure 1). Again, a low whole prostate dose was used; the baseline tumor control then was calculated to be 80.0%. Similarly to the previous scenario, the TCP-increase by a given SIB was also strongly related to the assumed sensitivity of the choline PET. Using a sensitivity of 81% the TCP was increased by 13.2% compared to 22.2% in the same setting employing the ASTRO definition. In contrast, for 64% sensitivity the increase was only 10.3%. Replacing the given α/β by higher values resulted in lower TCP gains. The lowest increase for TCP was seen for Valdagni's α/β with a low choline PET sensitivity: 9.0%.

**Table 7:** TCP-increase for different sensitivities of PET, low whole prostate dose, different α/β values, calculated α and low γ50(CN + 2 definition)

α [Gy^-1^]	α/β [Gy]	γ50	Det. rate PET [%]	Dose [Gy]	SIB [Gy]	Single dose [Gy]	TCP_conv _[%]	TCP Increase [%]
0.03	1.5	1.4	81	74	90	2	80	13.2
0.03	1.5	1.4	64	74	90	2	80	10.3
0.04	3.1	1.4	81	74	90	2	80	12.6
0.04	3.1	1.4	64	74	90	2	80	9.8
0.06	8.3	1.4	81	74	90	2	80	11.6
0.06	8.3	1.4	64	74	90	2	80	9.0

Calculation of the TCP-increase after boosting choline PET positive regions within the prostate up to 90 Gy. α/β was set to either Fowler's/Wang's or Valdagni's value, α was analytically determined in order to achieve agreement between calculated TCD50 and TCD50 obtained by Cheung et al. γ50 was again taken from Cheung's data (CN + 2 definition).

### 8. Different sensitivities of PET, high whole prostate dose, different α/β values, calculated α, low γ50 (CN + 2 definition)

Compared to Table 7 in Table 8 a higher whole prostate dose was used (78 Gy). The initial TCP could be improved to 80%. The increase in TCP was lower as it ranged from 9.1% (high detection rate of PET, low α/β) to 6.0% (low detection rate, high α/β).

**Table 8:** TCP-increase for different sensitivities of PET, high whole prostate dose, different α/β values, calculated α and low γ50(CN + 2 definition)

α [Gy^-1^]	α/β [Gy]	γ50	Det. rate PET [%]	Dose [Gy]	SIB [Gy]	Single dose [Gy]	TCP_conv _[%]	TCP Increase [%]
0.03	1.5	1.4	81	78	90	2	84.5	9.1
0.03	1.5	1.4	64	78	90	2	84.5	7.1
0.04	3.1	1.4	81	78	90	2	84.5	8.5
0.04	3.1	1.4	64	78	90	2	84.5	6.6
0.06	8.3	1.4	81	78	90	2	84.5	7.7
0.06	8.3	1.4	64	78	90	2	84.5	6.0

Calculation of the TCP-increase after boosting choline PET positive regions within the prostate up to 90 Gy, low homogeneous dose 78 Gy. α/β was set to either Fowler's/Wang's or Valdagni's result, α was analytically determined in order to achieve agreement between calculated TCD50 and TCD50 obtained by Cheung et al. γ50 was again taken from Cheung's data (CN + 2 definition).

## Discussion

Using a simplified mathematical model allowed us to determine the increase in TCP after an IMRT SIB based on choline PET positive intra-prostatic lesions. The model has been based on several fundamental assumptions including uniform clonogenic cell density, no interaction between adjacent tumor cells, no sub-volume effects and a uniform radio-sensitivity of all tumor cells. Furthermore the model does not consider population differences or time factors [61]. This model is substantiated by the fact that doses being needed to control microscopic tumor in an adjuvant/salvage setting seem to be almost as high as those used in primary therapy for macroscopic tumors [60].

It was shown that a SIB mediated increase of the given TCP is strongly dependent on the sensitivity of the choline PET, the γ50-value with special emphasis on the definition of tumor control, the dose used for the treatment of the whole organ and the α/β values.

We observed a high variation between the outcomes based on different initial assumptions. A critical limitation is the fact that there is no chance to derive α/β and α values for the calculation of dose-response relationships from the trial by Cheung et al. (the best data available to date) since a single fixed fractionation schedule was applied [35].

In keeping with this several inconsistencies occurred (Table 5, Table 6, Table 7 and Table 8): the calculated α values did not fit their counterpart in literature except for Fowler's data where the deviation was small. In this case the dependence on γ50 and the detection rate of choline PET became more important.

On the one hand, γ50 depends on the failure definition and data are different with longer follow-up data, and at present the confidence interval is still wide as γ50 = 2.2 [1.1-3.2, 95% CI] (ASTRO definition) or γ50 = 1.4 [0.2-2.5, 95% CI] (CN + 2 definition).

On the other hand, a study from Farsad et al. demonstrated that C-11-choline PET/CT has a relatively high rate of false-negative results on a sextant basis. In addition it has been clearly shown that non-malignant prostatic disorders may induce an increased ^11^C-choline uptake [62]. Our model calculations are not dependent on specificity as the irradiation of non-infiltrated voxels does not influence TCP but this will lead to unessentially big SIB target volumes.

Taken together, the relatively high efficacy rates of an IMRT based SIB are potentially overestimating the real benefit (Table 5, Table 6, Table 7 and Table 8, between 7.1% and 22.2%). Patient setup errors as well as intrafraction motion of the prostate were not considered throughout the whole estimation process which could potentially hamper the results in a negative way [63-67]. Another important factor influencing tumor control was neglected in the model: the risk of regional, i.e. pelvic nodal and/or systemic failure. This may be a potential source of limiting the effectiveness of this approach as it was assumed that local control entails biochemical control; in this regard a single cancer cell outside the prostate could violate this assumption and diminish tumor control.

Despite all of our considerations our model data are not in contrast to data provided by Kim et al. [68] claiming that selective boosting is more effective than homogeneous dose escalation as sparing of normal tissue is easier to achieve.

Furthermore, risk-adaptive optimization increases the therapeutic ratio as compared to conventional selective boosting IMRT. In another paper Kim et al. derive similar results, but mention the importance of the underlying imaging modality and consecutively their sensitivity in detecting occult tumor cells [69].

Utilizing an IMRT boost is an elegant technique but one has to mention another classical but suitable method: With brachytherapy the doses to the organs at risk are lower or similar to IMRT-only. Dose escalation for prostate tumors may also be easily achieved by brachytherapy alone [70].

## Conclusions

Regarding treatment planning in radiotherapy, choline PET may offer some advantages in terms of staging, tumor delineation and the description of biological processes. However, a TCP-increase related to any IMRT SIB on choline PET positive regions has to be considered as realistically low.

## Abbreviations

RT: radiotherapy; IMRT: intensity-modulated radiotherapy; SIB: simultaneous integrated boost; PTV: Planning target volume; LQ: linear-quadratic; TCP: tumor control probability; TCD50: tumor control dose 50%; PET: positron emission tomography; SUV: standardized uptake value; CI: confidence interval; SF: surviving fraction; EBRT: external beam radiotherapy; PSA: prostate-specific antigen; ASTRO: American Society for Therapeutic Radiology and Oncology; CN: current nadir; FWHM: full width at half maximum.

## Competing interests

The authors declare that they have no competing interests.

## Authors' contributions

MN developed the underlying mathematical model and wrote the manuscript. PB participated in the preparation of the manuscript. UG and CB provided the idea and participated in the conception as well as the preparation of the manuscript. All authors read and approved the final manuscript.

## Supplementary Material

Additional file 1 This file contains a sheet where parameters like choline PET sensitivity/specificity, α, α/β, γ50, TCD50, dose, SIB dose and single dose can be specified and a sheet carrying out all necessary calculation steps. Click here for file
